# Hæmoptysis

**Published:** 1894-01-27

**Authors:** 


### MEDICAL PROGRESS.

### HÆMOPTYSIS.

The remarks of Dr. T. H. Green[1] in his recent lecture on Hæmoptysis contain much that is valuable in dealing with this symptom.

On the question of the treatment of phthisical hæmorrhage, Dr. Green says truly that there is no class of cases in which more judgment is required, and few, perhaps, in which treatment is more apt to be empirical and unwise.

In every case of hæmoptysis, whether profuse or slight, the following medicinal treatment should be adopted: (1) First of all, endeavour to reassure your patient. Nothing is so valuable in quieting the patient and so favouring quiescence of the circulation, which tends to arrest the bleeding, as the assurance of the doctor. (2) Insist on complete rest. The patient should be left in bed in the semi-recumbent position, and if we know which is the diseased side, this is better uppermost. (3) The room should be kept cool, and free ventilation ensured. (4) The surface of the body should be kept warm—for this reason—that by keeping the surface warm you tend to dilate the superficial vessels, and so diminish the blood pressure in the internal organs. Especially take care to keep the extremities warm. Hot bottles to the feet and legs are often most useful in helping to arrest the bleeding. (5) The food must be reduced in quantity, given cold, and in a concentrated form. (6) Give the patient ice to suck. It relieves thirst and tends to check the cough.

As regards drugs, Dr. Green utters a warning against giving ergot because the patient has hæmoptysis. In all cases consider (*a*) the advisability of giving a purgative; (*b*) the advisability of checking the cough. A purgative is almost invariably called for; hydragogue purgatives are generally most suitable. It is often wise at the commencement of the attack to give a dose of calomel or of colocynth and blue pill, followed by a saline, or we may keep up a more regular action of the bowels by small doses of sulphate of magnesia with sulphuric acid, given more frequently. The judicious use of such remedies constitutes the most important part of the medicinal treatment of hæmoptysis.

The second point to consider is the relieving of the cough. A mucilaginous linctus, containing morphia, chloroform, and vin. ipecac., is usually the best for this purpose; a linctus or lozenge is better than attempting to check the cough by sedative inhalations.

When the hæmoptysis is slight no further treatment may be required; but if the loss of blood is considerable, we consider the advisability of giving some hæmostatic. Now, the exhibition of hæmostatics is the least important part of the treatment of hæmoptysis.

The hæmostatics which are used in the treatment of hæmoptysis fall into two groups—the styptics and the cardio-vascular depressants.

He does not recommend the use of styptics, but of much greater value are the hæmostatics which depress the heart and circulation—opium, tartar emetic, turpentine, nitrites, ipecacuanha, &c. These favour coagulation by diminishing the blood-pressure.

Of these OPIUM is by far the most important. Opium or morphia in moderate doses reduces the frequency and force of the heart and dilates the vessels. It thus tends to lower the blood-pressure, and by so doing favours that thrombosis at the seat of the rupture, which is the only means by which the hæmorrhage can be arrested. Not only so, but it quiets the restlessness of the patient, and checks the cough; and so, again, it is an eminently rational mode of treatment. Used with care and judgment, opium is, in Dr. Green's opinion, by far the most valuable remedy we possess in the treatment of dangerous (aneurismal) hæmoptysis. The best way to administer the drug is by hypodermic injection with morphia. About a quarter of a grain should be given at the outset of the attack, and it may be repeated if necessary. Obviously this treatment has its drawbacks. The morphia tends to interfere with the respiratory function; and when we remember that one of the dangers of profuse hæmoptysis is the coagulation of blood in the trachea and larger bronchi, we understand that we may dangerously interfere with the power to expectorate. Still, in spite of such disadvantages, this is by far the most valuable remedy we possess, and at the Brompton Hospital for Consumption the usual medicinal treatment of dangerous hæmoptysis is a purgative and a hypodermic dose of morphia.

He further alludes to certain other drugs which are used as hæmostatics in the treatment of hæmoptysis. Perhaps no drug is so largely used in the treatment of hæmoptysis as ERGOT. Dr. Green considers that the clinical evidence of its utility is extremely doubtful. It is supposed by some to be useful by acting as a styptic, contracting the small vessels, and in capillary hæmorrhage it may, perhaps, be so; but in the aneurismal bleeding any such explanation is untenable. Others say that ergot reduces blood-pressure by producing active dilatation of the veins. If this be so, its employment is rational. But he has himself lost all faith in ergot, and at Brompton the drug is rarely used. One of the physicians occasionally prescribes it in cases believed to be capillary.

Other remedies which have been used in hæmoptysis are turpentine and tartar emetic. The latter, at one time a favourite drug, is too powerful a depressant to be a safe one.

*The Nitrites.*—Nitrite of amyl and nitro-glycerine are very powerful dilators of the blood-vessels, and so valuable reducers of blood-pressure, but they accelerate the heart's action, and so are of doubtful utility in hæmoptysis. At the same time their employment is deserving of further trial, and nitro-glycerine given with some digitalis has sometimes appeared to be of use, the digitalis counteracting the accelerating action of the nitrite on the heart.

Venesection is a rational but heroic mode of treatment. When a patient is losing large quantities of blood from one of these aneurisms, the danger is due, not so much to loss of blood as to loss of blood in the wrong place, the blood coagulating in the air passages. One can understand that by opening a vein in the arm and withdrawing some blood, the blood-pressure might be reduced sufficiently to lead to the arrest of the bleeding from the ruptured aneurism. For obvious reasons such treatment can only in very exceptional cases be admissible.

Lastly, he adds a word on the use of the ICE-BAG. The application of an ice-bag to the chest in cases of hæmoptysis is now very common. Its utility, however, is extremely doubtful. It has this disadvantage—that by cooling the surface of the body it tends to increase the blood-pressure in internal organs, and it is difficult to conceive how an ice-bag applied over the chest could possibly influence bleeding from one of these aneurisms. He does not employ it himself, but if, when called to a case, finds an ice-bag on the patient's chest, and he likes it, does not advise its removal. The wise course to pursue is to be guided by the effect it has on the patient.

Comby[2] recommends blistering for the principal revulsant. This latter may be employed over the chest or, better still, over the back on both sides. As to medicines, he advises the employment of those agents that act upon the heart and vessels, and whose action is to modify their contraction. DIGITALIS, by regulating and slowing the cardiac movements, may modify or indirectly arrest hæmoptysis; QUININE, which contracts the small vessels, may be associated with digitalis, and especially in those cases that occur in malarial districts. Even ERGOT may be combined with the above remedies, as in the following prescription: ℞, powder of ergot, powder of digitalis, sulphate of quinine, of each, ten centigrammes; glycerin, sufficient quantity. Mix and make one pill.

One of these pills is given four or five times a day. The fresh powder of ergot may be given in doses of from one to two grammes in quassia, or in a sweetened mixture. Ergotine and ergotinine may also be employed.

Besides the above remedies, the author speaks well of TANNIN, also ACETATE OF LEAD, the extract of RHATANY (in quantities of from two to three grammes), and PERCHLORIDE OF IRON in the form of vapour. Acids may also be said to be effective as adjuvants. The same may be said of balsamic remedies, such as turpentine, terpine, and terpineol. TERPINE may be administered in cachets, each of these containing twenty centigrammes. Four or five cachets are given in the course of a day. IPECACUANHA is specially recommended in small as well as in large amounts, so as to produce either only nausea or violent vomiting. To allay pain, opium or morphine is to be relied upon.

Dr. W. R. Huggard[3], in a recent communication, records two cases in which the value of venesection in suitable cases of hæmoptysis is well shown.

His views are supported by Dr. Knowsley Sibley[4], who, after having treated several cases by morphine hypodermically, and having, unfortunately, seen a good many die in the way described, came to the conclusion that the line of treatment should be the opposite of this, and a stimulant should be administered, such as ether hypodermically, so as to give power to the organism to expel the blood by the mouth, and prevent it blocking up the lungs. Better results are to be obtained in this manner than by morphine. At the same time, it often struck him the most rational method would be to let blood escape from a vein in the arm, and in this way endeavour to prevent the almost inevitable asphyxia from the general inspiration of a large quantity of blood escaping into part of a diseased lung. Even if the hæmorrhage is not immediately fatal, blood clot in the healthy parts of the lungs is a very good soil for the rapid growth and spread of the tubercle bacilli, and so tends to convert what might be a chronic process into an acute one.

Dr. De Havilland Hall[5] calls attention to the value of IPECACUANHA powder in hæmoptysis of phthisis.

[1] Clin. Journ., Jan. 28, 1893. [2] Therap. Gaz. Extract La Méd. Mod., Nos. 45, 646, 1892. [3] Brit. Med. Journ., Jan. 28, 1893. [4] Ibid., Feb. 11, 1893. [5] Lancet, July 15.

